# Individual Monitoring of Immune Response in Atlantic Salmon *Salmo salar* following Experimental Infection with Infectious Salmon Anaemia Virus (ISAV)

**DOI:** 10.1371/journal.pone.0137767

**Published:** 2015-09-23

**Authors:** Bertrand Collet, Katy Urquhart, Milena Monte, Catherine Collins, Sandro Garcia Perez, Chris J. Secombes, Malcolm Hall

**Affiliations:** 1 Aquaculture and Fish Health, Marine Scotland, Aberdeen, Scotland, United Kingdom; 2 School of Biological Sciences, University of Aberdeen, Aberdeen, Scotland, United Kingdom; INRA, FRANCE

## Abstract

Monitoring the immune response in fish over the progression of a disease is traditionally carried out by experimental infection whereby animals are killed at regular intervals and samples taken. We describe here a novel approach to infectiology for salmonid fish where blood samples are collected repeatedly in a small group of PIT-tagged animals. This approach contributes to the reduction of animals used in research and to improved data quality. Two groups of 12 PIT-tagged Atlantic salmon (*Salmo salar*) were i.p infected with Infectious Salmon Anaemia Virus (ISAV) or culture medium and placed in 1 m^3^ tanks. Blood samples were collected at 0, 4, 8, 12, 16, 21 and 25 days post infection. The viral load, immune and stress response were determined in individual fish by real-time quantitative PCR (QPCR) on the blood cells, as well as the haematocrit used as an indicator of haemolysis, a clinical consequence of ISAV infection. “In-tank” anaesthesia was used in order to reduce the stress related to chase and netting prior to sampling. The data were analysed using a statistical approach which is novel with respect to its use in fish immunology. The repeated blood collection procedure did not induce stress response as measured by HSP70 and HSP90 gene expression in the un-infected animals. A strong increase in viraemia as well as a significant induction of Mx and γIP gene expression were observed in the infected group. Interleukin 10 was found induced at the later stage of the infection whereas no induction of CD8 or γ IFN could be detected. These results and the advantages of this approach are discussed.

## Introduction

Fish infectiology studies traditionally rely on the sequential sacrifice of animals and tissue sampling across the kinetics of infection [1,2]. Not only is this model costly in terms of the number of fish used, but it is based on the assumption that the animals are well characterised, genetically homogeneous and that the infection is synchronised between individuals. This latter statement applies reasonably well to inbred strains of rodents used for medical research that have a high phenotypic homogeneity [3,4]. However it is less applicable to fish, with the exception of zebrafish, since very few genetically homogeneous stocks such as inbred lines or clones are available, and when they do exist they are often associated with individual laboratories and are not maintained as a scientific resource for widespread use. Most fish utilised in such experiments are therefore originated from commercial farms or breeders. Furthermore, as poikilotherms, fish are highly sensitive to environmental or behavioural parameters.

Non-lethal methods based on biopsies have been used in the past with fish to measure the impact of environmental pollutants [5], stable isotopes [6] and pathogen load [7,8]. However, only a limited number of studies have attempted to investigate the immune relationship between host and pathogen at the individual host level [9,10]. Furthermore, no attempt has been made to describe the dynamic of infection and host response in the same individual throughout repeated non-lethal blood collections, an approach commonly used in veterinary studies with large terrestrial animals (rabbit, cow, sheep) and small rodents (rat, mouse) [11].

In the present study, we describe a novel design and analysis method for infectiology in Atlantic salmon *Salmo salar* whereby the level of gene expression in the blood cells was monitored over time from the same individually Passive Integrated Transponders (PIT)-tagged animals following experimental Infection with Infectious Salmon Anaemia Virus (ISAV). This new method has several advantages which include a reduction in the number of animals used and improved information around the understanding of variation in the individual response.

## Material and Methods

### Experimental design

This study was carried out in strict accordance with the UK Animals (Scientific Procedures) Act 1986 (ASPA) under the project licence PPL3965. The protocol was approved by the Marine Scotland Ethical Review Committee. All procedures were performed under MS222 anaesthesia, and all efforts were made to minimise suffering. Twenty four Atlantic salmon *Salmo salar* tagged with PIT were provided by Landcatch Natural Selection (Hendrix-Genetics), transported to the Level 3 Biosecurity Aquarium Facility at Marine Scotland and divided equally into two circular 1 m^3^ tanks. They were kept under natural photoperiod, sea water salinity 37 ‰ and at 10°C. They were fed once a day with pellets (EWOS). After a week of acclimation, all the fish were anaesthetised, weighed (average weight 423.1 ± 21.4 g), measured (average length 35.9 ± 0.6 cm) and injected intra-peritoneally with 100 μl culture medium (N = 12, 1 tank) or 100 μl ISAV Loch Nevis strain [12] containing 2.8 x10^6^ TCID_50_ (N = 12, 1 tank). Immediately before injection, a small blood sample (150 μl) was collected from the caudal vein. Subsequently, blood samples were collected at 4, 8, 12, 16, 21 and 25 days post infection (dpi). The total blood withdrawal was below 10% total blood volume as estimated as 5% of total body weight [13]. To minimise stress related to capture of animals and repeat handling, in-tank anaesthesia was carried out. The water was slowly drained to 500 L and 400 mL of MS222 (Sigma) at 50 mg/L in tap water was poured into the tank through the automatic feeder opening. After 2 min the animals were sufficiently sedated to allow sample collection and returned into a tank with fresh aerated seawater for recovery. The sampling for the 12 fish lasted less than 7 min in total. The blood was withdrawn from the caudal vein, in the sagittal plane with a 1 mL syringe (Beckman Dickinson) attached to a gauge 23 needle (BD).

The Haematocrit was measured within 1 hour of collection according to Billett [14]. Blood from the Haematocrit capillary was recovered using a syringe and combined with the remaining blood. The whole blood was centrifuged for 30 sec at 13,000 g at room temperature. The plasma was collected and stored at -80°C until processed. The remaining blood cells were vortexed, and 30 μl were collected and mixed with 300 μl RLT buffer (RNeasy kit, Qiagen, Crawley, UK) with 10% (v/v) β-mercapto-ethanol (Sigma) and stored at -80°C until processed. The remaining blood cells was stored at -80°C as backup material.

### RNA extraction, cDNA synthesis and QPCR gene-expression assays

Total RNA from blood cells was purified using a method modified from the RNeasy Mini kit (Qiagen). The mix of blood cells and RLTb was homogenised with a Tissue Lyser using one 5 mm stainless steel bead (Qiagen) for 1 min at 25 Hz at room temperature. The remaining steps in the procedure were carried out according to the manufacturer’s instructions (Qiagen RNeasy Mini kit method) and the RNA was eluted in 75 μl RNase-free water and stored at -80°C until use. RNA was reverse transcribed to cDNA using M-MuLV Reverse Transcriptase (New England Biolabs) using oligo-d(T)_16_ (Applied Biosystems) as follows: 8 μl of total RNA (approx. 0.5μg), 1μl 50 μM oligo-d(T)_16_, 1μl 10 mM dNTPs (Applied Biosystems), 2μl PCR water (Sigma-Aldrich) were mixed and heated to 65°C for 5 min and immediately chilled on ice. The final volume was adjusted to 20 μl by adding the following: Reverse Transcriptase buffer (50 mM Tris-HCl pH 8.3, 75 mM KCl, 3 mM MgCl_2_,), 10 mM DTT, 0.5 mM each dNTP, 0.4U RNase inhibitor (Applied Biosystems) and 200 Units M-MuLV Reverse Transcriptase. Reactions were incubated at 37°C for 90 min, heat inactivated at 95°C for 5 min, diluted 5 fold with water and finally stored at -80°C until further use. QPCR assays were performed on a LightCycler 480 system QPCR machine (Roche Applied Science) containing per reaction 4μl diluted cDNA.

Elongation factor α (ELFα) expression was used as an internal control to normalise gene expression levels across different samples. Taqman QPCR assays have been carried out according to [15] (ELFα, STAT2), [16] (STAT1), [17] (CD4, CD8, IL10), [18] (MX, γIP, IFNA, γIFN, IL1B) or using primers and probes given in Table 1 (STAT6, IL8, IL12A). QPCR assays for HSP70, HSP90, IL12A and STAT6 were carried out in a reaction containing 5.0 μl SYBRGreen Master Mix 1 (Roche Applied Science), 0.5 μM forward and reverse primer (sequence given in Table 1) in a total volume of 10 μl. QPCR cycling conditions were 95°C for 10 min followed by 45 cycles of 95°C for 30 sec, 62°C for 30 sec, 72°C for 45 sec. A melting curve was obtained by increasing the temperature to 99°C at a rate of 0.11°C/sec.

**Table 1:** Sequence and accession umbers related to the HSP70, HSP90, IL8, IL12A, IL4–13A and STAT6 primers used in this study.

Gene/Accession number	Forward primer (5'-3')	Reverse primer (5'-3')	Taqman probe	Size of product (bp)
Heat shock protein 90kDa alpha (cytosolic) hsp90ab1/ BT043623	TGCGCTACCACAGTTCTCAGTCC	GTCCTTGCTCTCGCCAGTG	-	114
Heat shock cognate 70 kDa protein putative/ BT059361	AACGTAACACCACCATCCCAACCA	GCCCTCTCACCCTCATACACCTG	-	101
Interleukin 8 IL8/ AGKD04024185	CCATTACTGAGGGGATGAGTCTGAGAGGC	GCTCAGAGTTGCAATGATCTCAGTGTCTCTGC	-	158
IL4–13A/ AGKD03034860	CACACACAATCTGACAGAGGATCTTCTGAG	CGATGCAGTATTGATGTTTGTTGTAAACCCTCAG		188
STAT6/ AGKD03023105	GGCACCCGGGAAACCAGTCCTCTTC	CGGAACCAAAAACGGAATCCTTTCCCCGG	-	426
Interleukine 12 α IL12A/ AGKD04000309	CCACATTCAGTGAGAGTGAGTGTCTGAGGAAC	GGGTCTGCAACATGTGAGGAAGGATCCCC	-	175

### Statistical Analysis

Real-time PCR Cp values for each molecular marker were standardised with respect to the efficiency of amplification of that marker and the amplification of the control housekeeping gene ELF resulting in an ‘expression value’. Expression values were then log transformed to improve their approximation to the normal distribution. The concept underlying the statistical analysis is to establish an ‘envelope’ for the unchallenged control group within which the majority of expression values for uninfected fish occur. Expression values for the challenged experimental group outside this envelope then represent a possible response to infection. There are several practical issues with implementing this including the possibility that expression of a molecular marker may occur at a level below the detection threshold of the QPCR assay, and the choice of an appropriate statistical test to facilitate inference of a difference in response between the unchallenged control and challenged experimental groups.

The possibility that a molecular marker may be expressed at a level below the assay detection threshold was evaluated by estimating the probability that one or more missing QPCR Cp values for a molecular marker in the unchallenged control group exceeded the QPCR Cp threshold (for this study 40). This was calculated by assuming that the Cp for a molecular marker in the unchallenged control group follow a normal distribution and that the Cp of any missing value exceeded 40. The mean and standard deviation of this assumed distribution was estimated from the ‘censored’ data by maximum likelihood and then used to estimate the probability that one or more of the missing values did indeed exceed the QPCR threshold. Probability values exceeding 0.05 were interpreted as indicating that censoring had occurred.

An envelope bounded by limits within which the majority of uninfected fish expression values occur was estimated from the 95 percentile interval (PI) of the unchallenged controls using the mean and standard deviation of the log expression values of this group. The mean and standard deviation of log expression values for molecular markers not affected by censoring were estimated using standard statistical methods and for potentially censored molecular markers were estimated by maximum likelihood assuming that all missing unchallenged control group expression values represented censored Cpabove the QPCR detection threshold. Log expression values for both the unchallenged control and challenged experimental groups were plotted together with an upper PI limit, the lower limit being included only for molecular markers not affected by censoring. The PI assume that there is no change in the mean or standard deviation of unchallenged control group expression values throughout the experiment.

It is very likely that some challenged experimental group expression values outwith the PI limits are false-positive and that some within are false-negative. To aid in the interpretation of these values the numbers of expression values occurring within and without the PI envelope for unchallenged control and challenged experimental groups are compared. A difference in response between the two groups is regarded as statistically significant if the p-value for an increase in the proportion of challenged experimental group expression values without the PI limits relative to the unchallenged control group is less than or equal to 0.05 as determined using a one-tailed Fisher’s Exact Test [19]. This approach assumes that the molecular response of infected animals is less constrained than that of uninfected animals. Although this test is relatively simple given the structure of the data our experience during analysis indicates that it performs more satisfactorily than other more sophisticated (and possibly more powerful) statistical approaches. P-values have not been modified to account for the analysis of multiple molecular markers and care should be taken not to over interpret ‘differences’ for suites of markers. Analyses were performed using the R statistical environment (version 3.1.2) [20] and supplementary R-packages VGAM (version 0.9–6) [21]. The script and data files are provided as supplementary information (S1 and S2 Files, respectively).

## Results

External ISA signs (lethargy, pale gills and haemorrhagic eyes) were visible in individuals F19 at day 21 and F22 at day 19. The following individuals were found dead or were killed when moribund: F22 at day 19, F19 at day 21, F24 at day 22, F15 and F23 at day 23. Remaining individuals were killed at day 25. Internal signs for ISA such as dark liver, enlarged spleen, ascites, petechial haemorrhaging of the visceral fat were visible in most of the infected individuals. Individuals from the control group showed no pathology.

**Fig 1:**
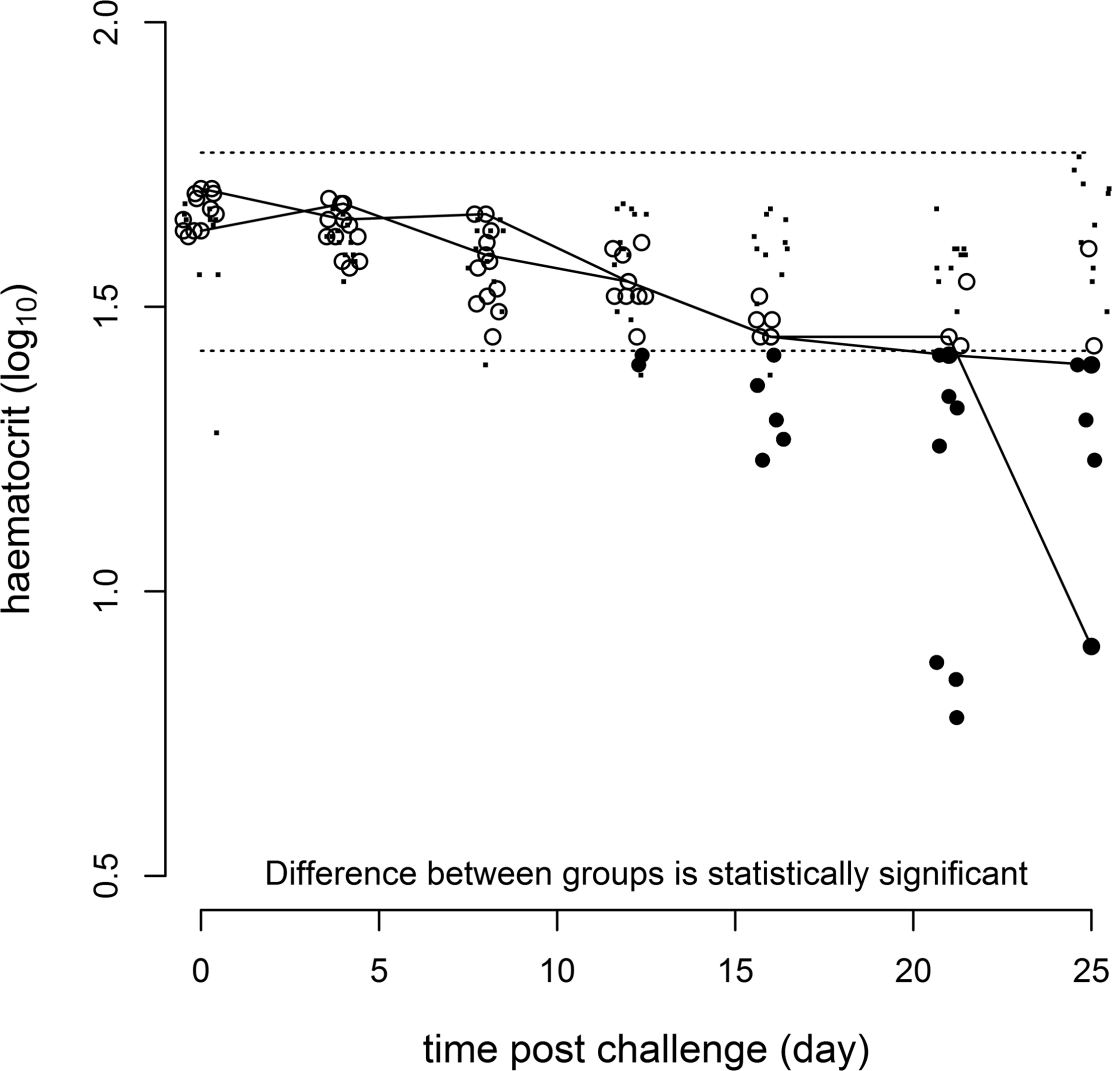
Response of log_10_ haematocrit over the course of the experiment. Dots (·) represent unchallenged control group values and the dashed lines (---) are the predicted 2.5 and 97.5 percentiles around these. Solid circles (•) represent challenged experimental group values which are regarded as responding and empty circles (o) challenged experimental group values which are regarded as not responding. The solid lines (—) are included as an aid to follow the response of those challenged experimental group individuals with the lowest and highest values conditional on representation at all time points. Values around the fixed time points of 0, 4, 8, 12, 16, 21 & 25 days have been horizontally jittered to improve visualisation.

The mean haematocrit for the uninfected control-group was 41.1±2.8% at the beginning of the experiment (dpi 0) and 45.4±2.9% at the termination of the experiment (DPI 25). There was no evidence of a consistent change in the haematocrit of the control-group throughout the experiment as statistically evaluated from a comparison of the residual variance of nested linear models [22] for haematocrit which included and excluded the explanatory variable of time. The mean haematocrit for the challenged experimental group was 46.7±1.0% at the beginning of the experiment and 23.1±3.7% at the termination of the experiment with evidence, using the statistical approach described in the materials and methods and presented in Fig 1 of a response to infection over time (p = 0.001).

**Fig 2:**
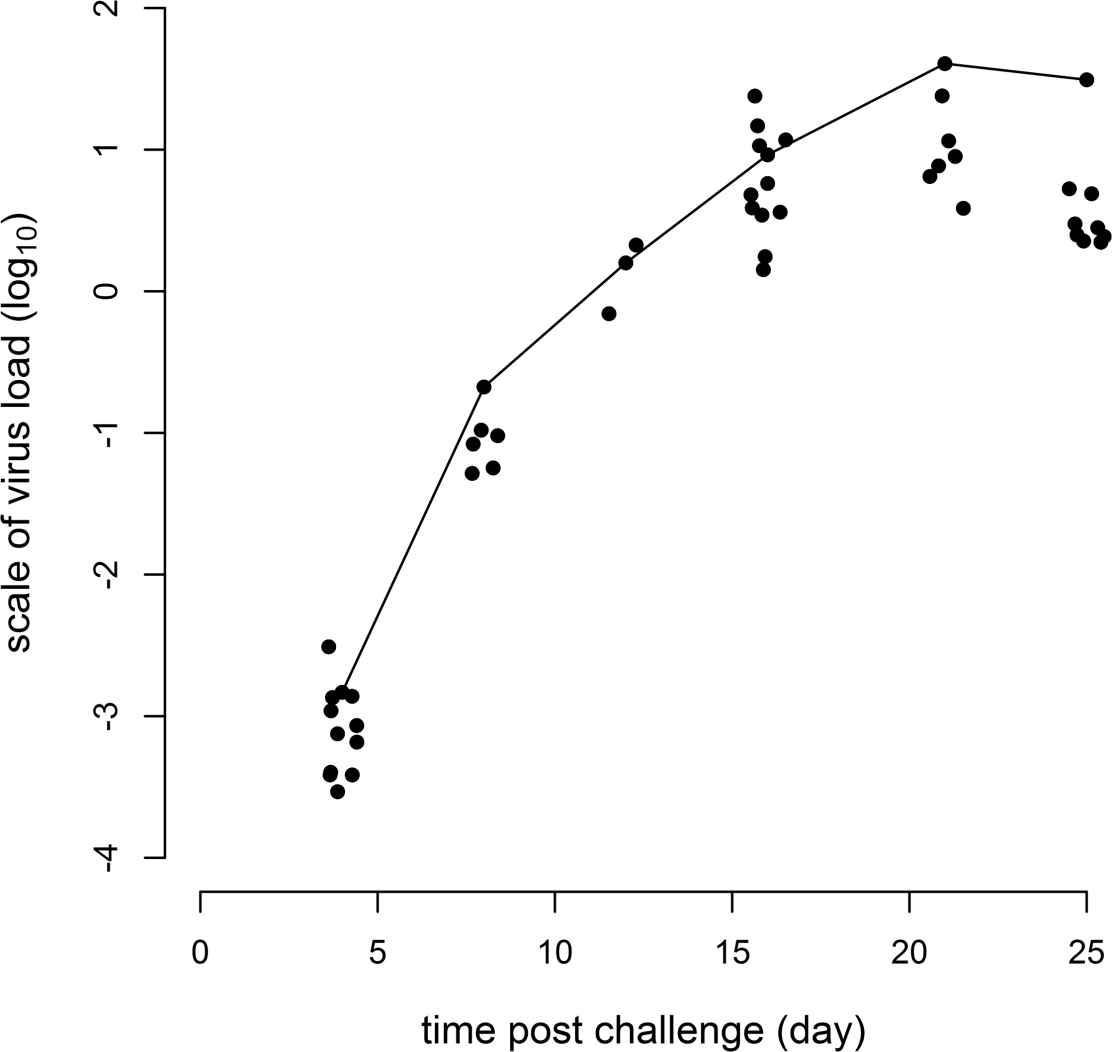
Log_10_ ISAV load for challenged experimental group individuals over the course of the experiment. The solid line (—) is included as an aid to follow the response of the individual with the highest value. Values around the fixed time points of 4, 8, 12, 16, 21 & 25 days have been horizontally jittered to improve visualisation.

Mean viraemia of the infected challenge-group based on QPCR and expressed as relative units, was 1x10^–3^ ± 1x10^–4^ at the first post-challenge sampling point (dpi 4) and 60 ± 30 at the termination of the experiment (dpi 25). A plot of post-challenge log response ratios shows an increase in viraemia over time with a plateau occurring towards the end of the experiment (Fig 2).

**Fig 3:**
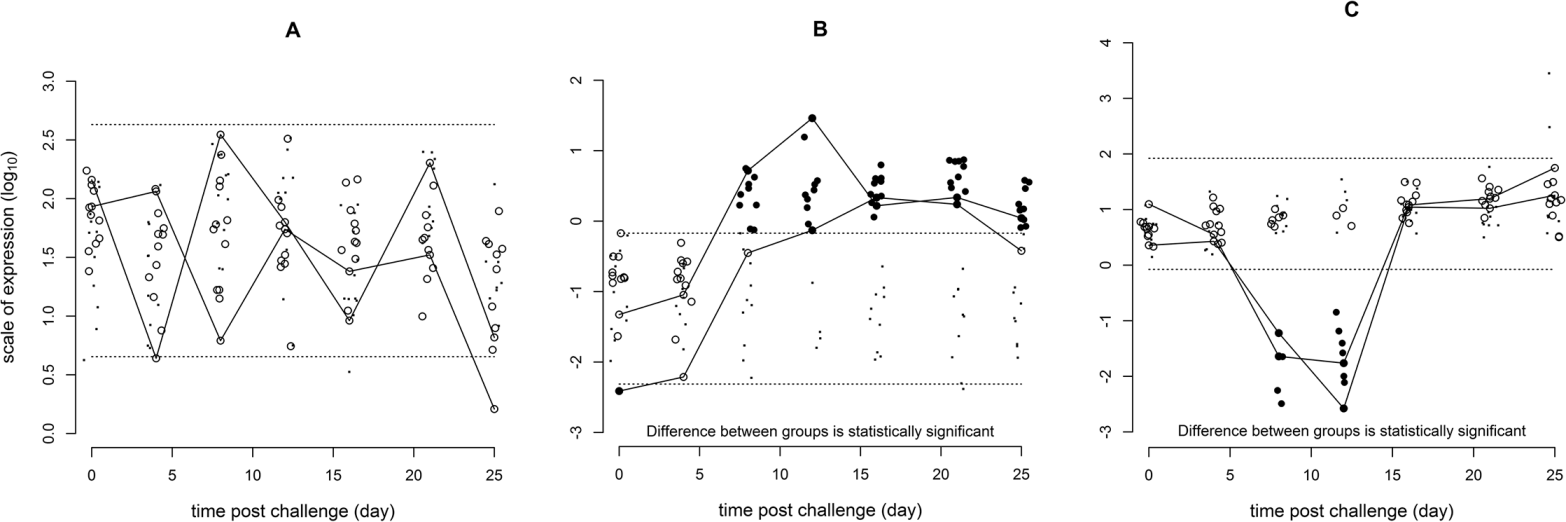
Plots illustrating different log_10_ response patterns. (A) No response for the immunological molecule IL8. (B) Sustained increase for the immunological molecule Mx. (C) Temporary decrease for the stress molecule hsp90a. Dots (·) represent unchallenged control group values and the dashed lines (---) are the predicted 2.5 and 97.5 percentiles around these. Solid circles (•) represent challenged experimental group values which are categorised as responding and empty circles (o) challenged experimental group values which are categorised as not responding. The solid lines (—) are included as an aid to follow the response of those challenged experimental group individuals with the lowest and highest values conditional on representation at all time points. Values around the fixed time points of 0, 4, 8, 12, 16, 21 & 25 days have been horizontally jittered to improve visualisation.

A summary of the host response to the challenge, with respect to analyses of a number of molecular markers, is presented in Table 2. There is evidence for a sustained increased response from 8 dpi onwards for the immunological markers Mx, STAT1 and γIP (e.g. Mx shown in Fig 3B). IL10 could be amplified only from samples collected from infected fish at the late stage of infection stage. An additional nine immunological markers (CD4, CD8, INFG, IFN1, STAT2, STAT6, IL8, IL12A and IL1B) were categorised as not responding to the challenge (e.g. IL8 shown in Fig 3A) although this does not preclude the possibility of some response below the statistical power of the experiment. The putative stress molecule HSP90a and HSP70 showed a temporary decrease at 8 and 12 dpi (e.g. HSP90a shown in Fig 3C). In addition there was no evidence of a consistent change in either stress molecule in the control-group over the time-course of the experiment as evaluated from a comparison of the residual variance of nested linear models for log response ratios which included and excluded the potential explanatory variable of time.

**Table 2:** Summary of results for immunological markers and haematocrit.

Marker	Response of challenge group	Control group censored
CD4	None	Yes
CD8	None	No
γIP	Increase	Yes
IFN1	None	No
γIFN	None	Yes
IL1B	None	No
IL8	None	No
IL10	Increase	Yes
IL12	None	No
Mx	Increase	No
STAT1	Increase	No
STAT2	None	No
STAT6	None	No
HSP70	Decrease	No
HSP90a	Decrease	No
Haematocrit	Decrease	No

**Fig 4:**
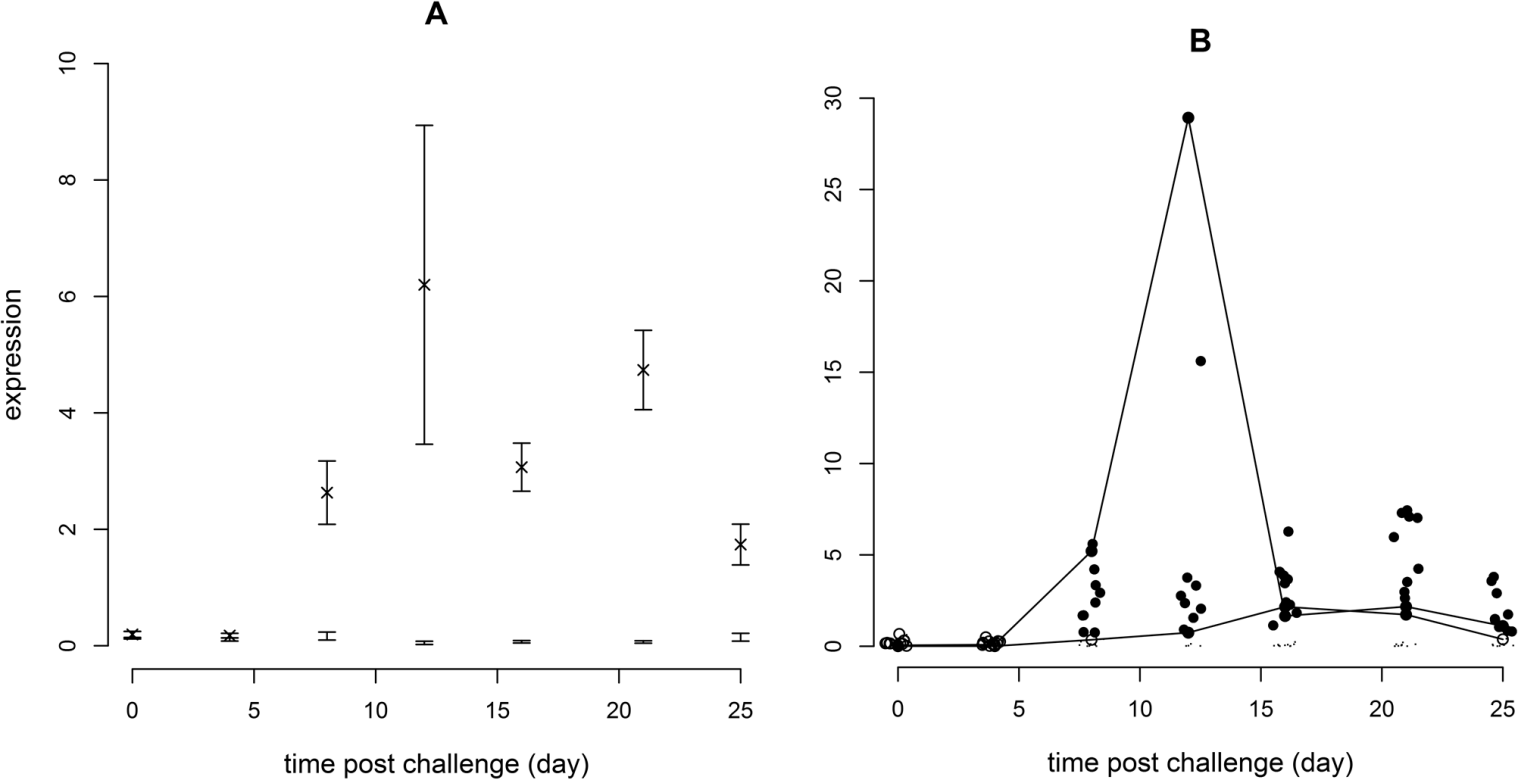
Response of Mx on the normal scale over the course of infection. (A) Analysis as performed in a typical published study. The symbol X represents the mean of challenged experimental group individuals at each time point with error bars representing the standard error of the mean assuming a normal distribution. Error bars without an X are for unchallenged control group values. (B) Analysis as described in this report. Dots (·) represent unchallenged control group values. Solid circles (•) represent challenged experimental group values which are categorised as responding and empty circles (o) challenged experimental group values which are categorised as not responding. The solid lines (—) are included as an aid to follow the response of those challenged experimental group individuals with the lowest and highest values conditional on representation at all time points. Expression values around the fixed time points of 0, 4, 8, 12, 16, 21 & 25 days have been horizontally jittered to improve visualisation.

Analyses of molecular responses in fish infectiology studies are usually made on the normal rather than the log scale involving the estimation and plotting of mean responses for challenge and control-groups at each time point sampled with the associated standard errors (Fig 4A). A plot of the individual response ratios for the challenge-group, categorised as responding or not-responding relative to the control-group (Fig 4B) demonstrates the statistically non-normal pattern of the individual expression values, and also shows that the response of challenge-group individuals may involve a small increase in value relative to the controls.

## Discussion

Although repeated blood collection is a common practice for large terrestrial veterinary animals, it is only rarely used in aquatic species for gene expression analysis, and to the authors’ knowledge has not been used to monitor the host immune response in fish [23,24]. The only record to date was a study by Raida et al., [10] where rainbow trout were infected with *Yersinia ruckeri* and a blood sample was collected just before infection. However, the design of this experiment was significantly different from natural rearing conditions since the animals were kept in individual tanks during the course of infection and only a single non-lethal sampling point (day 0) was collected and used as an individual reference point. Similarly, several non-lethal methods for the detection of pathogens or toxic compounds have been developed, but they were generally based on a single collection of blood [7] or by biopsy of gill [25,26], fin [8] or muscle [5] from valuable animals such as rare wild fish or broodstock. The nature of the biological response to infection with a live pathogen is characterised by a transient increase of cytokines in the plasma determined by the basal and peak levels and peak time [27]. As a consequence, the experimental design and in particular the sampling regime must allow sufficient resolution to describe the response kinetics and their inter-individual variation.

We describe the individual progression of the disease and immune response in a small group of Atlantic salmon following an experimental infection with a virulent strain of ISAV by intra-peritoneal injection. The present infectiology model can be referred to as “non-lethal” as opposed to the “lethal” model, currently in use by the large majority of scientists in the fish research community, where different individuals are killed for sampling at each time point during the course of an infection. The non-lethal sampling has been combined with the use of a more sophisticated statistical analysis than used in most fish experimental studies. This allows for a categorisation of when a fish responds following an initial standardisation of controls over time. The usual type of graph presented in piscine molecular immunological reports, as illustrated for the Mx gene (Fig 4A), confound the size of response for an individual with the timing of the response. Variation in expression values between individuals at each time point is also summarised as an error bar assuming a normal distribution and, with regard to statistical testing, assumes that sufficient individuals have been sampled to satisfy the central limit theorem. Neither do the mean and errors include individuals with expression values below the QPCR detection threshold thereby introducing a bias if such values occur. This new analysis (Fig 4B) provides an improved visualisation of the distribution of expression values and improved information on which of those are likely to be responding.

The advantage of using a non-lethal method is the ability to obtain data from the same individual allowing: i) estimates of inter-individual variability, ii) testing for the effect of pathogen infection or treatment on response levels and timing, and outcome of that response in an individual, and iii) potential reduction in the number of animals required for *in vivo* experiments. However, the model is limited to a minimal size of animals (150–200g for salmon) and is particularly relevant for molecular immune markers expressed in plasma or blood cells such as cytokines. The inter-individual variability is revealed itself by a proportion of animals responding to the pathogen. Furthermore, the maximal (sampled) amplitude in expression level is not synchronised in the responding animals. As a consequence, only a non-lethal model with individual repeated measurements can inform accurately on the onset and the intensity of a response, and help interpret robustly the resultant outcome of the response in relation to pathogen control. The data can be analysed using a statistical method which can infer whether a marker is statistically altered by the infection. Secondly, non-responder individuals can be identified and among the responders, differences in kinetics for the given parameter can be determined. However, because the kinetics of expression of the molecular markers are unknown, a sufficient frequency in the sampling regime is required to capture all changes and increase the likelihood to capture the true amplitude of the response.

The route and dose was similar to those used in a previous lethal ISAV infection [18]. In the present study the level of transcription of antiviral cytokines and related genes was measured in blood cells unlike the vast majority of previous studies where tissues are used [28]. Peripheral blood cells, when archived immediately after collection, include immune cells responsible for the production of some systemically released cytokines [29]. These cells also respond to cytokines by induction of downstream genes such as Mx1 or STAT1 as a response to type I IFN.

In the present study, 150 μl blood was collected 6 times non-lethally corresponding to a total of 5.4% of total blood volume as estimated by Gingerich et al., [30] withdrawn within a month. This sampling regime was not associated with any significant decrease in haematocrit in the control-group. An important contributor to stress in handling fish is the chase, capture and transfer of the animals by net in order to anaesthetise them [31–33]. A “in-tank-anaesthesia” method was built into the non-lethal design whereby the animals are left undisturbed until sedated by administration of the anaesthetic directly into the tank. For this purpose, the tank was required to be partially drained and several tests were performed to ensure that this operation did not alter the swimming behaviour of the animals above a certain volume threshold, contributing to a refinement of the animal procedures. If the approach described here is adopted more widely, these preliminary tests may need to be repeated for the aquarium system used in terms of tank diameter versus volume versus fish species. In this study there was also no evidence of a change in either haematocrit or either stress gene over the period of the experiment in the control fish. However, ISAV infection decreased significantly the haematocrit, reflecting the lysis of erythrocytes by the virus during infection [34].

A drawback of this method is the cost incurred by the larger quantity of anaesthetic required. In the longer term, data generated from the individual monitoring using this refined method is likely to prove more conclusive that those generated from the traditional lethal model and therefore fewer experiments would be required to answer a research question. The untreated control is used to give the basal line of immune parameters and providing these are stable over time, could be reduced in size. The non-lethal method is suitable for salmonids large enough to allow repeated blood collection, in the region of 100g.

A large number of animal experiments using lethal models are designed to verify induction or repression of newly discovered genes and to compare the levels of expression between different tissues of treated and un-treated animals [28]. The exclusive expression of a gene in a specific tissue provides information with regards to its function. However, this is usually carried out without perfusion of the tissues prior to sampling, resulting in the presence of blood cells in a quantity that can vary from one tissue to another. The anterior kidney, considered as a primary immune organ in fish, naturally contains a large proportion of blood that can affect the interpretation. It is therefore pertinent to investigate gene expression levels directly in the blood cells, to which the non-lethal model is restricted. In addition, a large number of immune parameters can be measured in small volume of plasma such as antibody levels, neutralisation activity of viraemia [35].

Viraemia can be monitored in the blood of infected fish. It has been demonstrated recently that ISAV targets erythrocytes [36–38]. The natural port of entry of ISAV is still unclear but it is thought that gills are the main organ of entry [39]. Although it has been reported that fish erythrocytes have a transcriptome it is unlikely to represent a major component of the transcriptome of total blood cells. ISAV have been shown to replicate effectively in leukocytes [40] suggesting that this category of blood cell is a potentially active producer of type I IFN when infected as reported in other vertebrate models [41]. It is nevertheless clear that ISAV binds to erythrocytes (haemadsorption) and in this way may distribute throughout the body to target cells such as endothelial cells [37]. This process ultimately results in the de-stabilisation of the erythrocyte membrane followed by its lysis and this is the main cause of the decrease in haematocrit. As the QPCR assay for ISAV used in this study does not distinguish between mRNA and the viral genome, the strong increase in ISAV gene expression could be explained by the production of new viral particles at replication sites in other tissues and their release in to the blood stream resulting in an increase in the viral load bound to erythrocytes.

A reduction in HSP70 and HSP90 expression at days 8 and 12 was observed. Cortisol is also known to suppress mRNA expression of HSP90 during cellular stress [42]. Therefore induction of stress and potentially circulating cortisol at onset of disease may have resulted in changes to HSP90 expression in blood cells. Though not demonstrated for ISAV specifically, HSP90 plays an essential role in the replication of orthomyxoviruses [43,44] and other viruses through stabilising the transcribed proteins. If the increase in viral transcripts observed in the blood reflected active replication, then suppression of HSP90 expression at early/intermediate stages of infection, 8–12 days post infection would not be expected. Higher levels of HSP90 and other heat shock proteins have been reported in tissues with early stage ISAV infections compared to late stage infections [45]. The difference in expression of HSP90 in blood versus other tissues perhaps indicates that replication occurs in the tissue cells and not in the blood. There is also a question of whether it is suppression of HSP90 in blood cells, or whether it is migration of those cells responsible for high transcriptomic activity from the blood to peripheral tissues which results in a decrease in expression e.g. leukocytes.

In a previous experiment using the same dose and infection route, STAT1 expression was found to be maximal 6 days after infection in the kidney tissue [16]. There was no significant increase in the level of STAT1 in the blood cells in the present experiment. It is possible that the basal level of STAT1 is higher in the blood cells than in kidney cells resulting in a reduced ability to detect a significant induction. The Mx gene was strongly induced in blood cells confirming its status as a marker for type I interferon and viral infection in fish [28]. Interleukin 10 transcripts were detected at the late stage of infection whereas no induction of CD8 or γIFN could be detected. This is in agreement with a mechanisms of viral infection reported in higher mammals whereby IL10 has a role in the inhibition of cytotoxicity [46, 47]. The existence of IL10 from viral origin suggests that this result may be an element of viral immuno-suppression [48].

An extension of the current work, comparing non-lethal blood sampling and lethal sampling of different tissues at the same time points to investigate host response, and to interpret its role in disease outcome, will further parameterise the usefulness of the non-lethal approach promoted here. Analysis of blood only will not be sufficient to provide understanding on the activation and role of specific immune effectors in different tissues, but it should enable the downstream resultant immune orientation, and its timing post infection, to be associated with disease outcome.

The stress response inherent to repeated anaesthesia and blood collection can be kept to a minimal level as demonstrated by this study, allowing a non-lethal infectiology model with a reduced number of animals to be used. The blood withdrawal did not affect the haematocrit over time in uninfected fish suggesting that the combination of volume of blood collected and frequency is not affecting fish welfare. The in-tank anaesthesia procedure reported here reduces stress related to chasing individual animals for sampling. Overall, the non-lethal model described can contribute to the reduction and refinement of animals used in experiments and generates improved information at the individual animal level. A statistical method provided as an R script (appendix A) can be used directly to analyse data generated by this model. This model can be immediately used in all functional genomics studies but has also the potential to be adapted to studies aimed at evaluating vaccine or treatment efficacy, in genetic selection breeding programs or in health monitoring programs in commercial farms. The analytical power of individual monitoring makes the non-lethal experimental model more valuable than the traditional destructive sampling method for a wide range of studies. The present study focused on an infection with a live viral pathogen. Real-time PCR allows us to measure the expression levels of pertinent genes in the blood cells that infer the early host response to a specific pathogen before the onset of any clinical signs. This can have application in monitoring programs where early prediction of an outbreak is critical. Studies on fish behaviour, toxicity, endocrinology, nutrition or physiology can also benefit and be refined if already used, from the described experimental design, method of sampling and data analysis. If adopted this would lead to a significant reduction in the use of animals in science combined with improvement in the data quality.

## Supporting Information

S1 File Script for R statistical package to perform the statistical analyses and to generate figures. Instruction and explanation are given in comments.
(TXT)

S2 File Excel spreadsheet giving the raw QPCR data (Cp values) and the Haematocrit levels for every samples. Instructions are given in the R script in S1.
(XLSX)

## References

[1] Turner PV, Brabb T, Pekow C, Vasbinder MA. Administration of Substances to Laboratory Animals: Routes of Administration and Factors to Consider. J Am Assoc Lab Anim Sci. 2011;50: 600–613. 22330705 PMC3189662

[2] Diehl K-H, Hull R, Morton D, Pfister R, Rabemampianina Y, Smith D, et al A Good Practice Guide to the Administration of Substances and Removal of Blood, Including Routes and Volumes. J Appl Toxicol. 2001;21: 15–23. 11180276 10.1002/jat.727

[3] Wiles S, Hanage WP, Frankel G, Robertson B. Opinion: Modelling infectious disease—time to think outside the box? Nat Rev Microbiol. 2006;4: 307–312. 16518420 10.1038/nrmicro1386

[4] Peters LL, Robledo RF, Bult CJ, Churchill GA, Paigen BJ, Svenson KL. The mouse as a model for human biology: a resource guide for complex trait analysis. Nat Rev Gen. 2007;8: 58–69. 10.1038/nrg2025 17173058

[5] Baker RF, Blanchfield PJ, Paterson MJ, Flett RJ, Wesson L. Evaluation of Nonlethal Methods for the Analysis of Mercury in Fish Tissue. Trans Am Fish Soc. 2004;133: 568–576.

[6] Kelly MH, Hagar WG, Jardine TD, Cunjak RA. Non-lethal Sampling of Sunfish and Slimy Sculpin for Stable Isotope Analysis: How Scale and Fin Tissue Compare with Muscle Tissue. North American J Fish Management 2006;26: 921–925.

[7] Bruno D, Collet B, Turnbull A, Kilburn R, Walker A, Gubbins M, et al Examination of diagnostic methods for *Renibacterium salmoninarum* causing bacterial kidney disease (BKD) in the UK. Aquaculture 2007;269: 114–122.

[8] Drennan JD, Lapatra SE, Samson CA, Ireland S, Eversman KF, Cain KD. Evaluation of lethal and non-lethal sampling methods for the detection of white sturgeon iridovirus infection in white sturgeon, *Acipenser transmontanus* (Richardson). J Fish Dis. 2007;30: 367–379. 17498180 10.1111/j.1365-2761.2007.00817.x

[9] Ellis AE, Cavaco A, Petrie A, Lockhart K, Snow M, Collet B. Histology, immunocytochemistry and qRT-PCR analysis of Atlantic salmon, *Salmo salar* L., post-smolts following infection with infectious pancreatic necrosis virus (IPNV). J Fish Dis. 2010;33: 803–818. 10.1111/j.1365-2761.2010.01174.x 20561142

[10] Raida MK, Holten-Andersen L, Buchmann K. Association between *Yersinia ruckeri* infection, cytokine expression and survival in rainbow trout (*Oncorhynchus mykiss*). Fish Shellfish Immunol. 2011;30: 1257–1264. 10.1016/j.fsi.2011.03.022 21501689

[11] Fluttert M, Dalm S, Oitzl MS. A refined method for sequential blood sampling by tail incision in rats. Lab Anim. 2000;34: 372–378. 11072857 10.1258/002367700780387714

[12] Murray AG, Smith RJ, Stagg RM. Shipping and the Spread of Infectious Salmon Anaemia in Scottish Aquaculture. Emerg Infect Dis. 2002;8: 1–5. 11749740 10.3201/eid0801.010144 PMC2730283

[13] Conte FP, Wagner HH, Harris TO. Measurement of blood volume in the fish (*Salmo gairdneri gairdneri*). Am J Physiol. 1963;205: 533–540. 14065906 10.1152/ajplegacy.1963.205.3.533

[14] Billett HH. Hemoglobin and Hematocrit In: Walker HK, Hall WD, Hurst JW, editors. Clinical Methods: The History, Physical, and Laboratory Examinations. Boston: Butterworths; 1990 pp 718–719. 21250045

[15] Collet B, Ganne G, Bird S, Collins C. Isolation and expression profile of a gene encoding for the Signal Transducer and Activator of Transcription STAT-2 in Atlantic salmon (*Salmo salar*). Dev Comp Immunol. 2009;33: 821–829. 10.1016/j.dci.2009.01.007 19428483

[16] Collet B, Bain N, Prevost S, Besinque G, McBeath A, Snow M, et al Atlantic salmon *Salmo salar* Signal Transducer and Activator of Transcription STAT-1: a contrasting kinetics of expression upon ISAV or IPNV infection. Fish Shellfish Immunol. 2008;25: 861–867. 10.1016/j.fsi.2008.01.011 18996723

[17] Collet B, Collins C. Comparative gene expression profile in two Atlantic salmon cell lines TO and SHK-1. Vet Immunol Immunopathol. 2009;130: 92–95. 10.1016/j.vetimm.2008.12.022 19162334

[18] McBeath AJA, Snow M, Secombes CJ, Ellis AE, Collet B. Expression kinetics of interferon and interferon-induced genes in Atlantic salmon following infection with IPNV and ISAV. Fish Shellfish Immunol. 2007;22: 230–241. 16806972 10.1016/j.fsi.2006.05.004

[19] Fisher RA. Statistical methods for research workers Edinburgh: Oliver & Boyd; 1934.

[20] R Core Team. R: a language and environment for statistical computing Vienna: R Foundation for Statistical Computing; 2014.

[21] Yee TW. The VGAM package for categorical data analysis. Journal of Statistical Software. 2010; 32(10): 1–34.

[22] Searle SR. Linear Models. New York: John Wiley and Sons; 1971.

[23] Steiner E, Balmelli C, Gerber H, Summerfield A, McCullough K. Cellular adaptive immune response against porcine circovirus type 2 in subclinically infected pigs. BMC Vet Res. 2009;5: 45 10.1186/1746-6148-5-45 20028550 PMC2806361

[24] Grant CFJ, Lefevre EA, Carr BV, Prentice H, Gubbins S, Pollard AJ, et al Assessment of T-dependent and T-independent immune responses in cattle using a B cell ELISPOT assay. Vet Res. 2012;43: 68 10.1186/1297-9716-43-68 23050495 PMC3487944

[25] Cornwell E, Bellmund C, Groocock G, Wong P, Hambury K, Getchell R, et al Fin and gill biopsies are effective non-lethal samples for detection of Viral haemorrhagic septicaemia virus genotype IVb. J Vet Diagn Invest. 2013;25: 203–209. 10.1177/1040638713476865 23404480

[26] McCormick SD. Method for nonlethal Gill Biopsy and measurement of Na^+^, K^+^-ATPase activity. Can J Fish Aquat Sci. 1993;50: 656–658.

[27] Paul WE, Seder RA. Lymphocyte responses and cytokines. Cell. 1994;76: 241–251. 7904900 10.1016/0092-8674(94)90332-8

[28] Collet B. Innate immune responses to viral infections of salmonid fish. Dev Comp Immunol. 2014;43: 160–173. 10.1016/j.dci.2013.08.017 23981327

[29] Baechler EC, Batliwalla FM, Karypis G, Gaffney PM, Moser K, Ortmann WA, et al Expression levels for many genes in human peripheral blood cells are highly sensitive to ex vivo incubation. Genes Immun. 2004;5: 347–353. 15175644 10.1038/sj.gene.6364098

[30] Gingerich WH, Pityer RA, Rach JJ. Estimates of plasma, packed cell and total blood volume in tissues of the rainbow trout (*Salmo gairdneri*). Comp Biochem Physiol. 1987;87: 251–256. 10.1016/0300-9629(87)90119-8 2886266

[31] Carragher JF, Sumpter JP. Corticosteroid Physiology in Fish In: Epple A, Scanes CG, Stetson MH, editors. Progress in Comparative Endocrinology. New-York: Wiley-Liss; 1990 pp 487–92. 2381951

[32] Klontz GW. Care of Fish in Biological Research. J Animal Sci. 1995;73: 3485–3492. 10.2527/1995.73113485x 8586609

[33] Wedemeyer G. Some Physiological Consequences of Handling Stress in the Juvenile Coho Salmon and Steelhead Trout. J Fisheries Res Board Canada. 1972;29: 1780–1783.

[34] Snow M, Raynard R, Bruno DW, van Nieuwstadt AP, Olesen NJ, Løvold T, et al Investigation into the susceptibility of saithe *Pollachius virens* to infectious salmon anaemia virus (ISAV) and their potential role as a vector for viral transmission. Dis Aquat Org. 2002;50: 13–18. 12152900 10.3354/dao050013

[35] Collet B, Urquhart K, Noguera P, Larsen K, Lester K, Smail D, et al A method to measure an indicator of viraemia in Atlantic salmon using a reporter cell line. J Virol Meth. 2013;191: 113–117. 10.1016/j.jviromet.2013.04.009 23602803

[36] Aamelfot M, Weli SC, Dale OB, Koppang EO, Falk K. Characterisation of a monoclonal antibody detecting Atlantic salmon endothelial and red blood cells, and its association with the infectious salmon anaemia virus cell receptor. J Anat. 2013;222: 547–557. 10.1111/joa.12033 23439106 PMC3633344

[37] Aamelfot M, Dale OB, Weli SC, Koppang EO, Falk K. Expression of the infectious salmon anaemia virus receptor on atlantic salmon endothelial cells correlates with the cell tropism of the virus. J Virol. 2012;86: 10571–10578. 10.1128/JVI.00047-12 22811536 PMC3457268

[38] Workenhe ST, Wadowska DW, Wright GM, Kibenge MJ, Kibenge FS. Demonstration of infectious salmon anaemia virus (ISAV) endocytosis in erythrocytes of Atlantic salmon. Virol J. 2007;4: 13 17254352 10.1186/1743-422X-4-13 PMC1793955

[39] Weli SC, Aamelfot M, Dale OB, Koppang EO, Falk K. Infectious salmon anaemia virus infection of Atlantic salmon gill epithelial cells. Virol J. 2013;10: 5 10.1186/1743-422X-10-5 23282149 PMC3560113

[40] Moneke E, Ikede BO, Kibenge FS. Viraemia during infectious salmon anaemia virus infection of Atlantic salmon is associated with replicating virus in leucocytes. Dis Aquat Org. 2005;5: 153–157. 10.3354/dao066153 16231641

[41] Siegal FP, Kadowaki N, Shodell M, Fitzgerald-Bocarsly PA, Shah K, Ho S, et al The Nature of the Principal Type 1 Interferon-Producing Cells in Human Blood. Science. 1999;284: 1835–1837. 10364556 10.1126/science.284.5421.1835

[42] Sathiyaa R, Campbell T, Vijayan MM. Cortisol modulates HSP90 mRNA expression in primary cultures of trout hepatocytes. Comp Biochem Physiol. 2001;129: 679–685. 10.1016/s1096-4959(01)00373-6 11399505

[43] Naito T, Momose F, Kawaguchi A, Nagata K. Involvement of Hsp90 in assembly and nuclear import of influenza virus RNA polymerase subunits. J Virol. 2007;81: 1339–1349. 17121807 10.1128/JVI.01917-06 PMC1797515

[44] Chase G, Deng T, Fodor E, Leung BW, Mayer D, Schwemmle M, et al Hsp90 inhibitors reduce influenza virus replication in cell culture. Virology. 2008;377: 431–439. 10.1016/j.virol.2008.04.040 18570972

[45] Jørgensen SM, Afanasyev S, Krasnov A. Gene expression analyses in Atlantic salmon challenged with infectious salmon anaemia virus reveal differences between individuals with early, intermediate and late mortality. BMC Genomics. 2008;9: 179 10.1186/1471-2164-9-179 18423000 PMC2387173

[46] Ejrnaes M, Filippi CM, Martinic MM, Ling EM, Togher LM, Crotty S, et al Resolution of a chronic viral infection after interleukin-10 receptor blockade. J Exp Med. 2006;203: 2461–2472. 17030951 10.1084/jem.20061462 PMC2118120

[47] Tsai T-T, Chuang Y-J, Lin Y-S, Wan S-W, Chen C-L, Lin C-F. An emerging role for the anti-inflammatory cytokine interleukin-10 in dengue virus infection. J Biomed Sci. 2013;20:40 10.1186/1423-0127-20-40 23800014 PMC3700829

[48] Ouyang P, Rakus K, van Beurden SJ, Westphal AH, Davison AJ, Gatherer D, et al IL-10 encoded by viruses: a remarkable example of independent acquisition of a cellular gene by viruses and its subsequent evolution in the viral genome. J. Gen. Virol. 2014;95: 245–262. 10.1099/vir.0.058966-0 24225498

